# Kidney double positive T cells have distinct characteristics in normal and diseased kidneys

**DOI:** 10.1038/s41598-024-54956-3

**Published:** 2024-02-23

**Authors:** Sanjeev Noel, Andrea Newman-Rivera, Kyungho Lee, Sepideh Gharaie, Shishir Patel, Nirmish Singla, Hamid Rabb

**Affiliations:** 1 Department of Medicine, Johns Hopkins University, Ross 970, 720 Rutland Avenue, Baltimore, MD 21205 USA; 2 Department of Urology, Johns Hopkins University, Baltimore, MD USA

**Keywords:** Double positive T cells, AKI, DPT cells, Ischemia reperfusion, Cisplatin, Nephrology, Kidney diseases, Lymphocytes

## Abstract

Multiple types of T cells have been described and assigned pathophysiologic functions in the kidneys. However, the existence and functions of TCR+CD4+CD8+ (double positive; DP) T cells are understudied in normal and diseased murine and human kidneys. We studied kidney DPT cells in mice at baseline and after ischemia reperfusion (IR) and cisplatin injury. Additionally, effects of viral infection and gut microbiota were studied. Human kidneys from patients with renal cell carcinoma were evaluated. Our results demonstrate that DPT cells expressing CD4 and CD8 co-receptors constitute a minor T cell population in mouse kidneys. DPT cells had significant Ki67 and PD1 expression, effector/central memory phenotype, proinflammatory cytokine (IFNγ, TNFα and IL-17) and metabolic marker (GLUT1, HKII, CPT1a and pS6) expression at baseline. IR, cisplatin and viral infection elevated DPT cell proportions, and induced distinct functional and metabolic changes. scRNA-seq analysis showed increased expression of *Klf2* and *Ccr7* and enrichment of TNFα and oxidative phosphorylation related genes in DPT cells. DPT cells constituted a minor population in both normal and cancer portion of human kidneys. In conclusion, DPT cells constitute a small population of mouse and human kidney T cells with distinct inflammatory and metabolic profile at baseline and following kidney injury.

## Introduction

T cells are important constituents of the adaptive immune system required to elicit highly specific inflammatory responses towards a large number of stimuli, including viral and bacterial pathogens, parasitic nematodes, and sterile inflammation^1–4^. Immature T cell precursors (i.e., lymphoid progenitors) produced in the bone marrow migrate to the thymus to undergo further developmental differentiation and functional maturation^5^. During thymic development immature T cells transition from a double negative (DN; lacking CD4 and CD8 co-receptors) to a double positive (DP) stage before acquiring a terminally stable CD4+ or CD8+ single positive (SP) stage. Subsequently, these terminally differentiated CD4+ and CD8+T cells migrate from the thymus to peripheral organs, such as kidneys, in response to various stimuli and migratory cues.

T cells are present in both healthy and diseased kidneys and have been implicated in various kidney diseases including allograft rejection^6–8^, interstitial nephritis^9^, glomerulonephritis^10,11^, ischemic and nephrotoxic AKI^12,13^, viral infections^14^ and gut dysbiosis^15,16^. The normal adult C57BL/6 J mouse kidney has CD4+ (~ 45%), CD8+ (~ 35%) and DN (~ 15%) T cells^17^. Similarly, normal human kidney is known to harbor CD4+ (~ 35%) and CD8+ (~ 45%) T cells in addition to DNT (~ 10%) cells^18,19^. These T cell subsets have been studied in depth in AKI. For example, CD4+T cells play a pathogenic role in ischemic and nephrotoxic AKI^12,13,20^. Additional studies showed that subsets of CD4+T cells called T helper 17 (Th17) cells have pathogenic roles whereas regulatory T cells (Tregs) mediate injury resolution and improve tissue recovery post- injury^21–23^. CD8+T cells are present in normal kidneys of mice and humans and their elevated proportion in kidney transplant patients is associated with a higher risk of graft dysfunction^24–26^. More recent studies demonstrate the presence of immunoregulatory double-negative (DN) T cells in normal, AKI and lupus-involved kidneys^17,18,27,28^.

In addition to these well-studied T cell subsets, several recent studies in both mice and humans have described DPT cells, expressing CD4 and CD8 co-receptors^29–31^. However, since DPT cells are found in very small numbers, they remain understudied both in the normal and diseased kidneys. Additionally, their cellular heterogeneity and functional characteristics remain unclear. We sought to study DPT cells in normal mouse kidneys and in models of ischemic and cisplatin-induced AKI, AKI to CKD, viral infection and germ-free conditions. DPT cells were also studied in human kidneys obtained from renal cell carcinoma patients in the presence or absence of background end-stage kidney disease (ESKD).

## Results

### DPT cells are present in normal mouse kidney, and each cell expresses both CD4 and CD8 co-receptors

**Figure 1:**
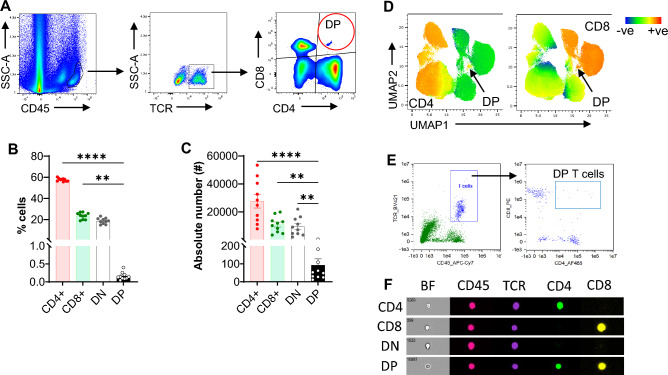
Kidney DPT cell constitute a minor population in normal mouse kidney and expresses CD4 and CD8 co-receptors on individual cells. (**A**) Representative flow images showing the gating strategy used to identify DPT cells among CD45+ KMNCs. A total of 1 × 10^6^ KMNCs were stained with anti-CD45, TCR, CD4 and CD8 antibodies and acquired on Cytek Aurora. CD45+ cells were gated and doublets removed using SSC-H versus SSC-W and FSC-H versus FSC-W parameters. Subsequently, Zombie NIR positive dead cells were removed and single live cell population was gated for TCRβ to identify T cells. T cells were then gated for CD4 and CD8 markers to identify different T cell subsets. DPT cells, shown in red circle, were positive for both CD4 and CD8 markers. (**B**) Graph showing the percentage of DPT cells along with CD4, CD8 and DNT cells in normal mouse kidney (n = 10). (**C**) Graph showing the absolute numbers of CD4, CD8, DN and DPT cells in normal kidney (n = 10). (**D**) UMAPs showing expression of CD4 (left UMAP) and CD8 (right UMAP) co-receptors in CD45+TCR+ cells. (**E**) Dot plots generated using IDEAS software for analyzing AMNIS Imagestream flow data showing the gating strategy used to identify DPT cells (right panel) among kidney T cell population (left panel). (**F**) Panel showing expression of CD45, TCR, CD4, and CD8 on individual CD4, CD8, DN, and DPT cells. Brightfield images on the leftmost column show individual CD4, CD8, DN, and DPT cells. Data are expressed as mean ± sem and compared by non-parametric Kruskal–Wallis test followed by Dunn’s post-hoc analysis. * = *P* ≤ 0.05, ** = *P* ≤ 0.01, **** = *P* ≤ 0.0001.

We first assessed the percentage and absolute number of DPT cells in normal mouse kidneys. DPT cells were identified from KMNCs as CD45+ cells expressing TCRβ and CD4 and CD8 co-receptors per the gating strategy shown in Fig. 1A. DPT cells constituted 0.16 ± 0.03% of T cells in normal mouse kidney, which was significantly less than the CD4 (57.64 ± 0.47%; *P* < 0.0001), CD8 (23.54 ± 0.97%; *P* = 0.001) and DN (18.66 ± 0.87%; *P* = 0.09) T cells (Fig. 1B). There were significantly fewer absolute numbers of DPT cells than CD4, CD8 and DNT cells (Fig. 1C). Evaluation of splenic T cells showed a very small percentage (0.07 ± 0.01%) and absolute number of DPT cells, compared to CD4, CD8 and DNT cells, demonstrating that DPT cells also constitute a minor population of total T cells in lymphoid tissue (Supplemental Fig. 1A and B). Although, the percentage of DPT cells was significantly higher in the kidney, the absolute numbers were much higher in the spleen (Supplemental Fig. 1C and D). We next carried out machine learning with an unbiased uniform manifold approximation and projection (UMAP) analysis of CD45+TCR+ kidney cells to confirm the existence of DP cells in mouse kidneys. UMAP analysis also identified a very small population of cells expressing both CD4 and CD8 co-receptors (Fig. 1D). To further ascertain that DPT cells express both CD4 and CD8 co-receptors on individual cells and to rule out the possibility of CD4-CD8 doublets, we performed flow cytometry-based imaging of individual kidney DPT cells using AMNIS Imagestream Imaging Flow Cytometer. Doublets were carefully removed from the analysis using brightfield “area” versus “aspect ratio” of the cells. Single cells were identified based on intermediate area value and a high aspect ratio (Supplemental Fig. 1E). As shown in Fig. 1E and F, both CD4 and CD8 co-receptors were expressed on individual DPT cells, further confirming their existence in the kidney.

### Human kidneys have DPT cells

**Figure 2:**
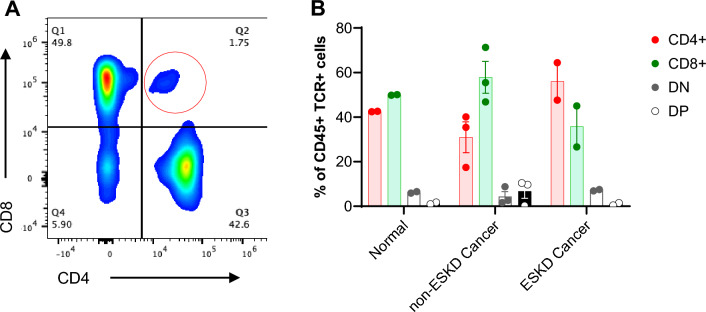
DPT cells in normal and cancer portion of kidney nephrectomies from renal cell carcinoma (RCC) patients. (**A**) Representative flow plot showing DPT cells in human kidneys. The gating strategy for human kidneys was identical to mouse kidneys shown in Fig. 1A. A total of 7 human kidney samples were analyzed, consisting of 2 from the adjacent “normal” portion, 2 from the cancer portion of RCC patients with ESRD and 3 samples from RCC patients without ESKD. (**B**) Graph showing percent DPT cells in normal, non-ESKD cancer and ESKD cancer tissue compared to CD4, CD8 and DNT cells. Data are expressed as median (IQR) and compared by non-parametric Kruskal–Wallis test followed by Dunn’s post-hoc analysis. * = *P* ≤ 0.05, ** = *P* ≤ 0.01, *** = *P* ≤ 0.001, **** = *P* ≤ 0.0001. BF- Brightfield.

Next, we evaluated human kidney tissue to see whether DPT cells are present in human kidneys. We procured human kidney tissue from adjacent “normal” and cancer portion of the nephrectomies from renal cell carcinoma (RCC) patients that either had ESKD (n = 2) or did not have ESKD (n = 3). We found DPT cells in both the normal (0.97 (0.44–1.49)%) and the cancer portion of the nephrectomies. Similar to the mouse kidneys, DPT cells constituted a minor population compared to CD4, CD8 and DNT cells (Fig. 2A and B). Kidney tissue from non-ESKD cancer had the greater median percentage (9.69 (0.29–10.40)%) of DPT cells compared to kidneys with ESKD cancer (1.35 (0.95–1.75)%).

### Kidney DPT cells have a distinct activation, proliferation and memory phenotype

**Figure 3:**
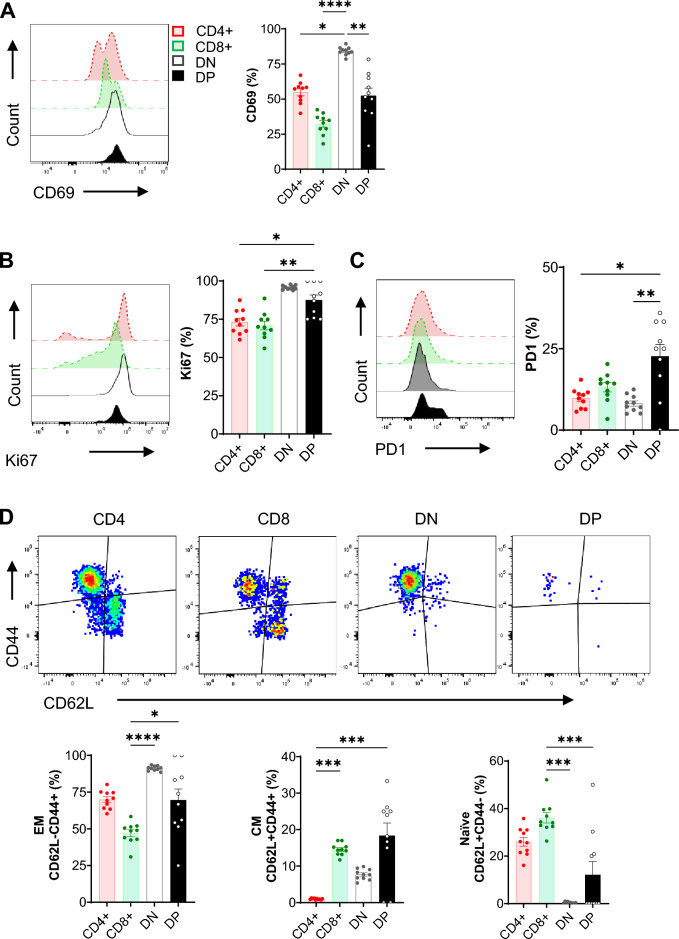
Kidney DPT cells have an activated and effector/central memory phenotype at baseline. A total of 1 × 10^6^ KMNCs were stained with anti-CD45, TCR, CD4, CD8, CD69, PD1, CD62L and CD44 and intracellular Ki67 antibodies. Graphs showing the percentage of (**A**) CD69, (**B**) Ki67 and (**C**) PD1 positive kidney CD4, CD8, DN and DPT cells in normal mouse kidneys (n = 10). (**D**) CD62L and CD44 markers were used to identify basal levels of naïve (CD62L+CD44−), effector memory (EM; CD62L−CD44+) and central memory (CM; CD62L+CD44 +) CD4, CD8, DN and DPT cells in normal mouse kidneys (n = 10). Data are expressed as mean ± sem and compared by non-parametric Kruskal–Wallis test followed by Dunn’s post-hoc analysis. * = *P* ≤ 0.05, ** = *P* ≤ 0.01, *** = *P* ≤ 0.001.

We next characterized murine kidney DPT cell activation status (CD69), proliferation (Ki67), and memory status (CD62L and CD44) at steady state (Fig. 3). DPT cells had significantly less CD69 (52.37 ± 5.44%) expression compared to DNT cells (84.19 ± 0.94%, *P* = 0.005) though it was comparable to both CD4 (54.81 ± 2.45%) and CD8 (32.32 ± 2.36%) T cells. Additionally, the percentage of CD69+DN T cells was significantly higher than CD4 (*P* = 0.03) and CD8 (*P* ≤ 0.0001) T cells (Fig. 3A). However, kidney DPT cells had significantly higher Ki67 expression (87.45 ± 3.3%) compared to CD4 (73.11 ± 2.55%, *P* = 0.03) and CD8 (70.84 ± 2.81%, *P* = 0.009) T cells but was comparable to DN (95.69 ± 0.44%) T cells (Fig. 3B). Kidney DPT cells also had a significant expression of immune checkpoint molecule PD1 (22.66 ± 3.64%) compared to CD4 (9.81 ± 0.96%, *P* = 0.02) and DN (8.14 ± 0.80%, *P* = 0.002) T cells (Fig. 3C). We further evaluated CD62L and CD44 expression to ascertain the memory status of kidney DPT cells. 69.56 ± 7.48% kidney DPT cells had an effector memory phenotype (EM; CD62L−CD44+) followed by 18.33 ± 3.48% central memory (CM; CD62L+CD44+) and 12.1 ± 5.56% naïve (CD62L+CD44−) cells. Additionally, DN T cells had significantly higher percentage of EM cells (*P* ≤ 0.0001) and reduced naïve cells (*P* ≤ 0.0001) compared to CD8 T cells. We also found increased percentage of CM cells in CD8 subset compared to CD4 (*P* ≤ 0.001) subset (Fig. 3D). Comparison of kidney and splenic DPT cells showed comparable CD69 but increased Ki67expression in kidney cells compared to splenic DPT cells (Supplemental Fig. 1E and F).

### Kidney DPT cells produce proinflammatory cytokines

**Figure 4:**
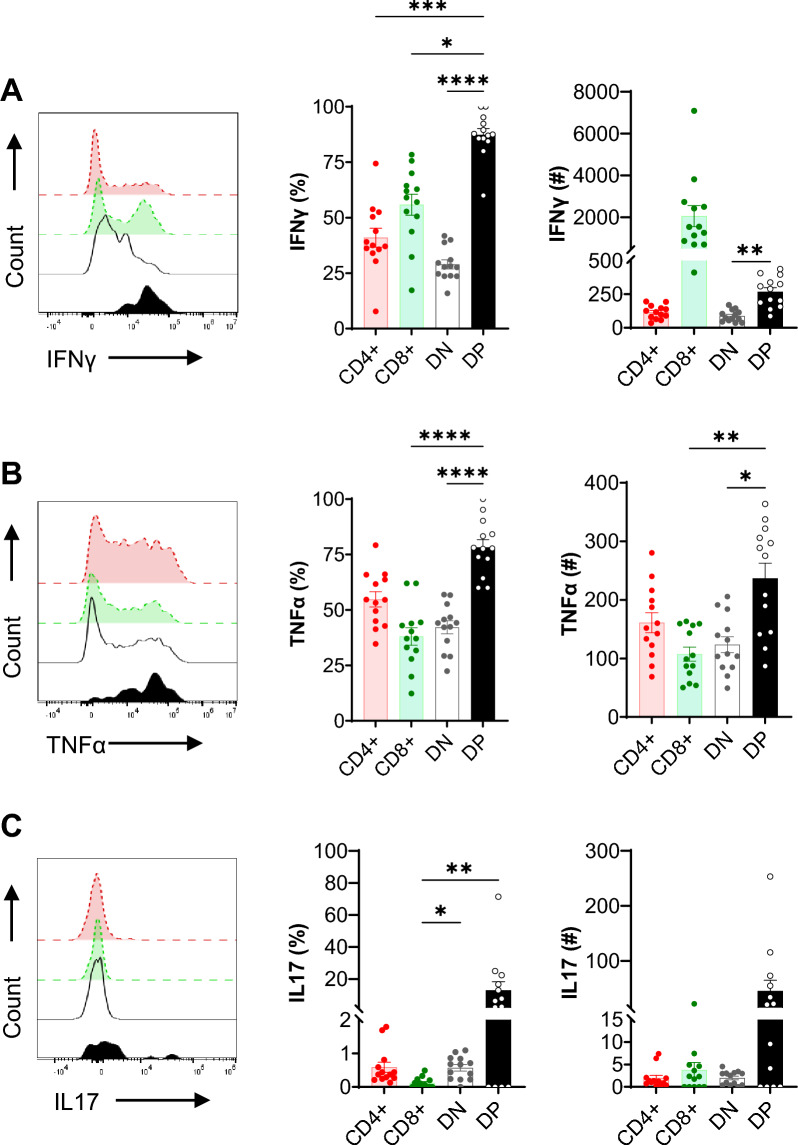
Kidney DPT cells produce proinflammatory cytokines at baseline. A total of 1 × 10^6^ KMNCs were incubated with T cell activation cocktail containing PMA/Ionomycin/Brefeldin A for 4 h at 37 °C. Cells were then stained with Zombie NIR followed by surface markers (CD45, TCR, CD4, CD8) and intracellular cytokine (IFNγ, TNFα and IL-17) antibodies. Percentage (left graph) and absolute numbers (right graph) of (**A**) IFNγ, (**B**) TNFα and (**C**) IL-17 expressing DPT cells in normal mouse kidney (n = 13). Data are expressed as mean ± sem and compared by non-parametric Kruskal–Wallis test followed by Dunn’s post-hoc analysis. * = *P* ≤ 0.05, ** = *P* ≤ 0.01, *** = *P* ≤ 0.001, **** = *P* ≤ 0.0001.

We then evaluated kidney DPT cell expression of inflammatory cytokines at steady state. The percentage of DPT cells that were IFNγ positive (87.32 ± 2.80%) was significantly (*P* < 0.01) higher than CD4 (40.98 ± 4.28%), CD8 (55.84 ± 4.73%) and DN (28.96 ± 2.07%) T subsets (Fig. 4A). Similarly, the percentage of DPT cells positive for TNFα (78.28 ± 3.46%) was significantly (*P* < 0.01) higher than CD8 (38.05 ± 3.93%) and DN (42.18 ± 2.94%) T subsets (Fig. 4B). Furthermore, the percentage of IL-17 positive DPT cells was significantly (*P* < 0.01) higher than the CD8 T cells (12.87 ± 5.44% vs 0.12 ± 0.04%) (Fig. 4C). Despite the small numbers of DPT cells present in the kidney, the absolute numbers of IFNγ and TNFα positive DPT cells were significantly higher than DNT (IFNγ and TNFα) and CD8 T cells (TNFα). Furthermore, the majority of DP T cells were positive for both IFNγ and TNFα expression suggesting that these cells express multiple cytokines (Supplemental Fig. 1H). Kidney DPT cells had increased IFNγ expression compared to splenic DPT cells (Supplemental Fig. 1I–L).

### DPT cells have a distinct metabolic profile in normal kidney

**Figure 5:**
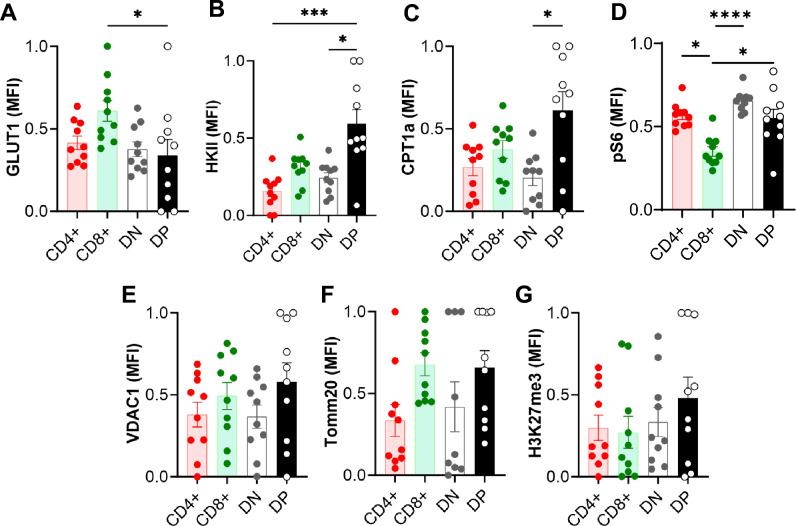
Kidney DPT cell have a distinct basal metabolic profile compared to CD4, CD8 and DNT cells. (**A**–**G**) A total of 1 × 10^6^ KMNCs were stained with Zombie NIR followed by surface markers (CD45, TCR, CD4, CD8) and intracellular markers (GLUT1, HKII, CPT1a, pS6, VDAC1,  TOMM20 and H3K27me3) to assess DPT cell metabolism (n = 10). DPT cells had a distinct metabolic profile that was intermediate between CD4 and CD8 T cells. Data are expressed as mean ± sem and compared by non-parametric Kruskal–Wallis test followed by Dunn’s post-hoc analysis. * = *P* ≤ 0.05, ** = *P* ≤ 0.01.

We assessed several key proteins involved in cellular energetics and metabolic pathways to evaluate basal DPT cell metabolic status using flow cytometry. The normalized mean fluorescent intensity (MFI) of glucose transporter protein GLUT1 in kidney DPT cells (0.34 ± 0.10) was comparable to CD4 (0.42 ± 0.04) and DN (0.38 ± 0.04) T cells but significantly lower compared to CD8 (0.61 ± 0.06; *P* = 0.02) T cells (Fig. 5A). However, the expression of glycolysis rate-limiting protein hexokinase II (HKII) was higher in DPT cells (0.60 ± 0.10) compared to CD4 (0.16 ± 0.04, *P* = 0.0001) and DN (0.24 ± 0.03, *P* = 0.01) T cells (Fig. 5B). Additionally, the expression of carnitine palmitoyltransferase I (CPT1a), a mitochondrial membrane protein involved in fatty acid metabolism, was significantly higher in DPT cells compared to DN (0.61 ± 0.11 vs 0.20 ± 0.05, *P* = 0.02) T cells (Fig. 5C). Furthermore, the expression of mTOR signaling related ribosomal protein S6 (pS6) was significantly higher in kidney DPT cells compared to CD8 (0.50 ± 0.09 vs 0.20 ± 0.04, *P* = 0.03) T cells (Fig. 5D). There was no difference in the expression of VDAC1, Tomm20 and H3K27me3 between DP and other T cell subsets (Fig. 5E–G).

### Kidney DPT cells increase following cisplatin treatment and viral infection

**Figure 6:**
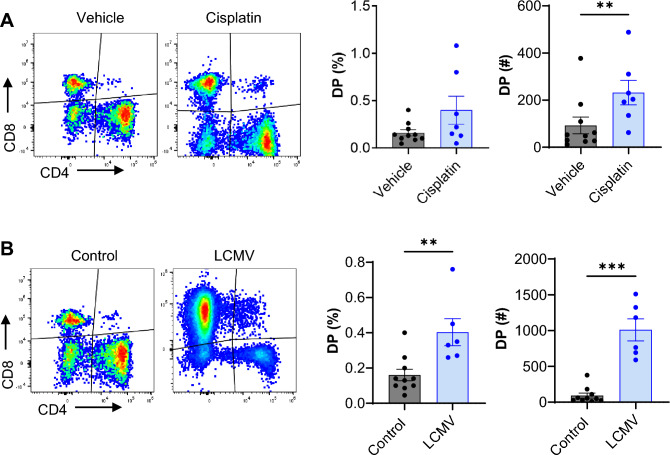
Kidney DPT cell proportion increase after nephrotoxic injury and viral infection. (**A**) Percentage (left graph) and absolute numbers (right graph) of DPT cells in vehicle (n = 10) and cisplatin treated (n = 7) mice at 72 h timepoint. (**B**) Percentage (left graph) and absolute numbers (right graph) of DPT cells in control (n = 10) and LCMV infected (n = 6) mice, 1 week after infection. Data are expressed as mean ± sem and compared by non-parametric Kruskal–Wallis test followed by Dunn’s post-hoc analysis. ** = *P* ≤ 0.01, *** = *P* ≤ 0.001.

To evaluate kidney DP cell response during kidney diseases we studied cisplatin-induced AKI and a viral infection model. Cisplatin, a chemotherapeutic agent is commonly used for treating multiple types of cancer and is a major cause of nephrotoxic AKI clinically. WT mice were treated with a single cisplatin injection (25 mg/Kg) and the effect on DPT cell proportion and function was evaluated. We observed significant increase in DPT cell absolute numbers (cisplatin 231.8 ± 51.72 vs control 92.50 ± 35.44, *P* ≤ 0.01) and an increasing trend in the percentage (cisplatin 0.40 ± 0.14% vs control 0.16 ± 0.03%, *P* = 0.17) in cisplatin treated mice compared to vehicle control mice at 72 h (Fig. 6A). Given the increased DPT cell proportion after cisplatin injury, we next evaluated the proliferation and activation status of DPT cells by assessing Ki67 and CD69 expression. We found no difference in either CD69 or Ki67 expression in DPT cells from cisplatin treated or vehicle control mice (Supplemental Fig. 2A and B). However, PD1 expression was significantly increased in DPT cells in cisplatin treated mice compared to vehicle control mice at 72 h (Supplemental Fig. 2C). Furthermore, there was no change in EM, CM or naïve DPT cells after cisplatin treatment (Supplemental Fig. 2D–F). Evaluation of metabolic markers showed no specific change except in pS6, which declined after cisplatin treatment (Supplemental Fig. 2G–L).

We next evaluated how kidney DPT cells respond to systemic lymphocytic choriomeningitis virus (LCMV) infection. LCMV infection model is a standard approach to evaluate stimulated T cell responses in mice^32^. For these studies, WT mice were infected with LCMV and kidney DPT cells quantified after 1 week post infection. We found a significant increase in the percentage (0.40 ± 0.08% vs 0.16 ± 0.03%, *P* < 0.01) and the absolute numbers (1010 ± 154 vs 92 ± 35.44, *P* < 0.01) of DPT cells in LCMV infected mice compared to control (Fig. 6B). Although we did not observe changes in the percentage of CD69 and Ki67 expressing DPT cells between the groups, LCMV infected mice showed a significant increase in the absolute numbers of CD69+ and Ki67+DPT cells compared to control mice (Supplemental Fig. 3A and B). There was also increased expression of immune checkpoint molecule PD1 in LCMV infected mice compared to control mice. LCMV infection resulted in increased expression of metabolic markers GLUT1, TOMM20, pS6 and CPT1a in DPT cells (Supplemental Fig. 3C).

### Kidney DPT cells are not affected by gut microbiota

To further assess the role of the microbiome on kidney DPT cells, we evaluated DPT from kidneys of germ-free (GF) mice housed in a specialized facility compared to conventional mice^33^. We found no difference in either the percentage or the absolute numbers of DPT cells between GF and the WT mice. Additionally, no difference was observed in DPT cell activation, memory status or cytokine production between GF and WT mice (Supplemental Fig. 4).

### Kidney DPT cells proportion increase in ischemic AKI with attenuated cytokine production

**Figure 7:**
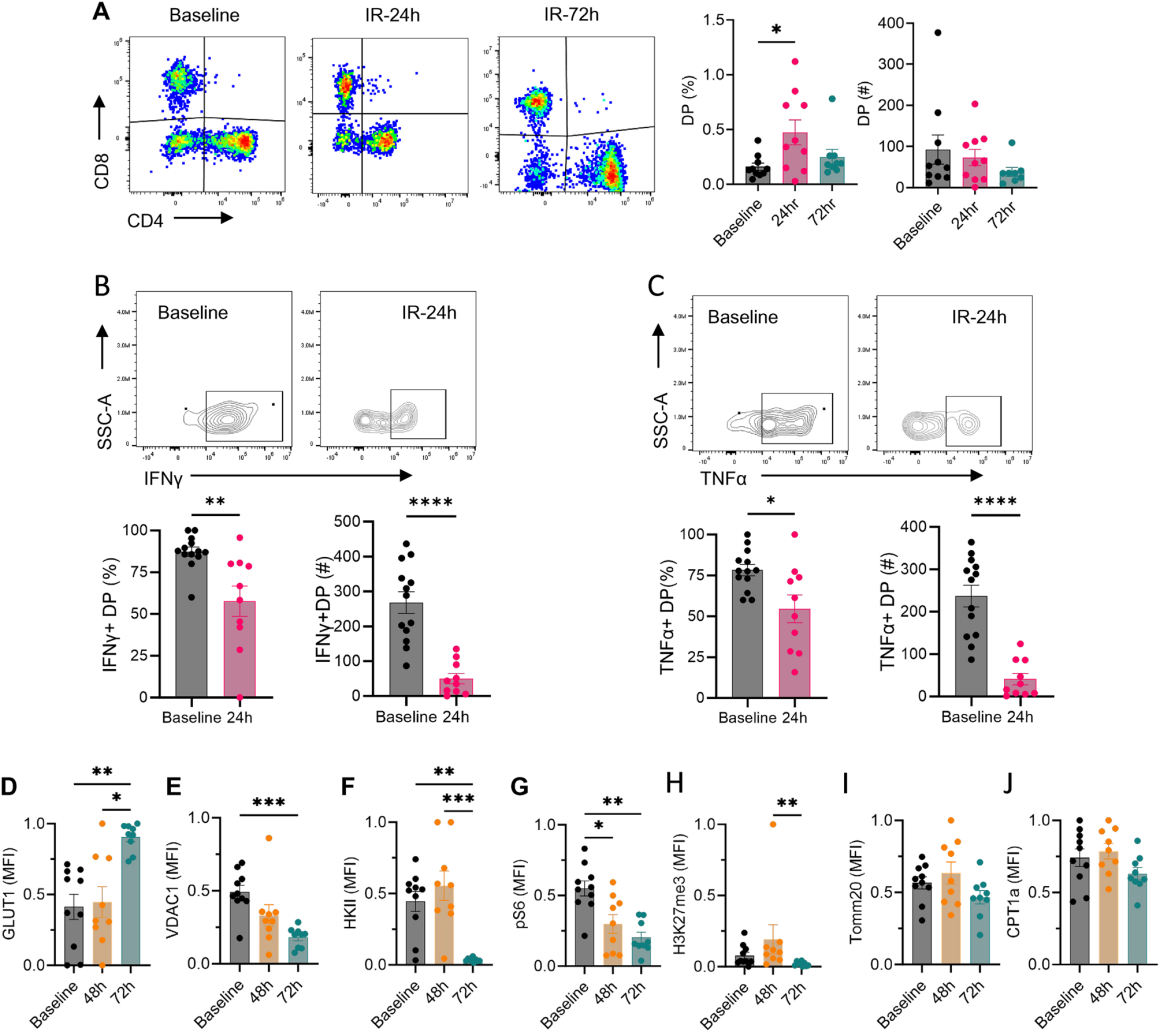
Ischemia reperfusion injury increases kidney DPT cell percentage and alters their cytokine and metabolic profile. (**A**) Percentage (left graph) and absolute numbers (right graph) of DPT cells in WT mice subjected to 27 min bilateral ischemia reperfusion injury and euthanized either after 24 h (n = 10) or 72 h (n = 9). Mice were euthanized under the effect of Ketamine/xylazine as described in the methods section. Baseline levels of DPT cells were quantified from normal non-surgery mouse kidneys (n = 10). (**B** and **C**) KMNCs from 24 h post-IR mice (n = 10) and control baseline (n = 13) mice were isolated and assessed for cytokine expression. The percentage (left panel) and absolute number (right panel) of IFNγ and TNFα positive DPT cells decreased after IR injury. (**D**–**J**) Separate group of mice were used to study effect of IR injury on metabolic markers and euthanized at either 48 h (n = 9) or 72 h post-IR (n = 9). IR injury increased GLUT1 expression but VDAC1, HKII, pS6 and H3K27me3 were reduced compared to baseline. Data are expressed as mean ± sem and compared by non-parametric Kruskal–Wallis test followed by Dunn’s post-hoc analysis. * = *P* ≤ 0.05, ** = *P* ≤ 0.01, *** = *P* ≤ 0.001, **** = *P* ≤ 0.0001.

We next evaluated the effect of IR injury on kidney DPT cells. For these studies WT mice underwent bilateral kidney IR injury followed by an assessment of DPT cell proportion and functional changes. We found increased DPT cell percentage at 24 h after IR injury compared to baseline (0.47 ± 0.11% vs 0.16 ± 0.03%, *P* = 0.048) which returned closer to basal levels by 72 h (0.25 ± 0.07%). However, absolute numbers of DPT cells did not change at 24 h (73.16 ± 19.51) and 72 h (38.28 ± 10.83) time points post-IR compared to baseline (92.50 ± 35.44) (Fig. 7A). Furthermore, the percentage and absolute numbers of IFNγ and TNFα producing DPT cells reduced significantly, 24 h post-IR compared to baseline (Fig. 7B and C respectively). Assessment of metabolic markers after IR injury showed increased expression of GLUT1 at 72 h, whereas VDAC1, HKII, pS6 and H3K27me3 were significantly reduced after IR injury (Fig. 7D–J). Evaluation of the long-term effect of IR injury on kidney DPT cells in a unilateral severe IR model of AKI to CKD progression showed no difference in either the proportion or the cytokine expression between injured and contralateral kidneys at 4 weeks after IR injury (Supplemental Fig. 5).

### scRNA-seq analysis of kidney DPT cells

**Figure 8:**
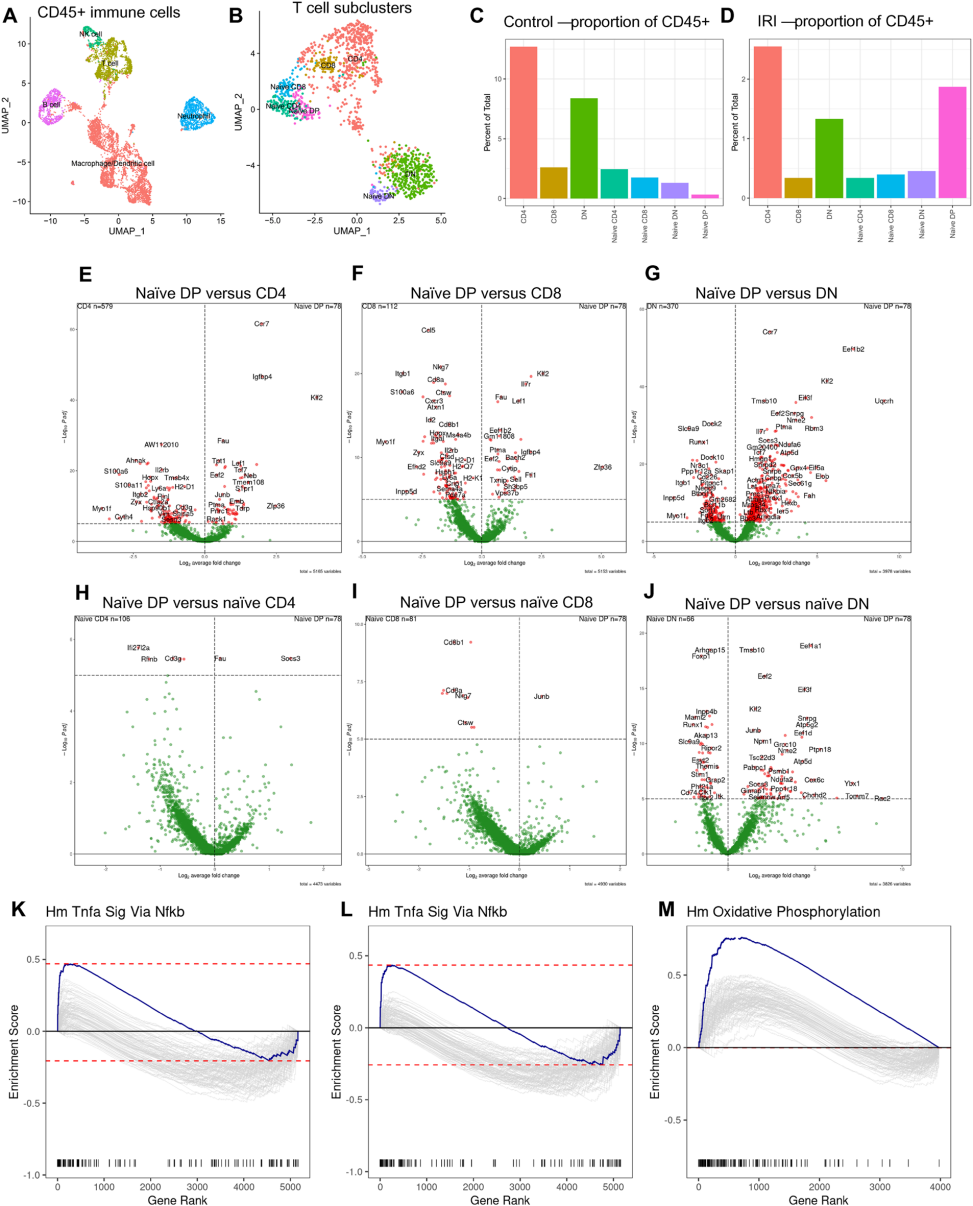
scRNA-Seq analysis of DPT cells at baseline and 24 h post-IR kidneys of WT mice. CD45+ cells were flow sorted from pooled KMNCs (n = 5/group) and 10,000 cells targeted for 10X Chromium-based encapsulation. (**A**) UMAP showing major immune cell populations identified in the scRNA-seq data. (**B**) UMAP showing different T cell populations and the presence of naive DPT cells in WT kidneys. (**C** and **D**) Plots showing DPT cell proportions in control and post-IR kidneys. (**E–G**) Volcano plots showing top differentially expressed genes (DEGs) in naïve DPT cells compared to effector CD4, CD8 and DNT cells, respectively. (**H**–**J**) Volcano plots showing top differentially expressed genes (DEGs) in naïve DPT cells compared to naive CD4, CD8 and DNT cells. (**K**, **L**) Gene set enrichment analysis (GSEA) of differentially expressed genes (DEGs) in showing increased enrichment of TNFα signaling in DPT cells compared to CD4 and CD8 cells and (**M**) increased OXPHOS compared to DNT cells.

To further elucidate similarities and differences between DPT cells compared to CD4, CD8 and DNT cells and to understand their roles during AKI, we reanalyzed recently published scRNA-seq data from CD45+cells that were flow sorted from control and post-IR kidneys (https://zenodo.org/record/7314511)^34^. We identified all major immune cells, including T cells, in our scRNA-seq dataset (Fig. 8A). Further analysis of T cell cluster showed a small but distinct naïve DPT cell cluster (*Sell, Tcf7, Il7r, Cd4* and *Cd8* expressing cells) along with naïve and effector populations for CD4, CD8 and DNT cells (Fig. 8B). There were greater number of DPT cells from the ischemic AKI kidneys (66/78) than control kidneys further confirming our flow data (Fig. 8C and D). Differential expression analysis showed several highly variable genes in DPT cells compared to effector CD4, CD8 and DNT cells (Fig. 8E–G). Particularly, the expression of *Klf2* in DPT cells was high compared to CD4, CD8 and DNT cells. Furthermore, *Ccr7* was highly expressed in DPT cells compared to CD4 and DNT cells. We found very few differentially expressed genes when comparing naïve DPT cells with naïve CD4 and CD8 T cells however, there were several differentially expressed genes between naïve DPT and naïve DNT cells including *Klf2* (Fig. 8H–J). Exploration of transcriptomic data using gene set enrichment analysis (GSEA) showed enrichment of TNFα signaling related genes in DPT cells compared to CD4 and CD8 T cells. In contrast, genes related to OXPHOS were enriched in comparison to DNT cells (Fig. 8K–M).

## Discussion

This study demonstrates that DPT cells are present in the mouse and the human kidneys and constitute a minor population of total T cells. Single cell flow analysis by AMNIS confirmed that these are truly dual receptor expressing T cells and not doublet artifacts on flow cytometry. Kidney DPT cells have an effector/central memory phenotype and produce proinflammatory cytokines in normal mouse kidneys. Kidney DPT cells increase after ischemic and cisplatin induced AKI, as well as viral infection in WT mouse kidneys with distinct functional and metabolic changes; however, they are not influenced by a germ-free environment. Single cell sequencing demonstrates a unique transcriptional profile at baseline and in AKI kidneys.

DPT cells were previously thought to be found only in the thymus as they are an intermediate stage between the DN and SP stages during T cell development^35^. Recent observations show that DPT cells are present in the non-lymphoid tissues including human kidney cancer tissue^29,30^. The origin of kidney DPT cells in this study is unclear, both at baseline and after injury, especially since the expression of CD4 and CD8 co-receptors is under very strict transcriptional regulation^35^. However, based on existing evidences from in vivo studies, terminally differentiated effector CD4+T cells could acquire the α-chain that is normally found on CD8+ cells. Alternatively, a mechanism similar to in vitro cytokine-induced CD4 expression on CD8+ T cells could also explain DPT cells in the kidneys. Defective positive thymic selection, that may lead to retention of both CD4 and CD8 co-receptors on the developing T cell, is also speculated to result in peripheral DPT cells^30,36^. Peripheral DP T cells are heterogenous population and generally subdivided into cells expressing CD4hi and CD8α, CD8αβ and CD4lo, and CD4hi and CD8αβhi. Additionally, terminally differentiated CD4+ T cells may also express CD8αα^30,37^. Our flow analysis used antibody targeting CD8α chain and thus cannot conclude if these cells were only CD8αα or CD8αβ.

We found that kidney DPT cells were active and produced proinflammatory cytokines at baseline but post-ischemic DPT cells showed an attenuated proinflammatory cytokine expression, suggesting reduced cytotoxic/proinflammatory properties following IR injury. A previous ex vivo study on peripheral blood DPT cells from healthy individuals found a memory phenotype and expression of chemokine receptors (e.g., CCR7, CXCR3, CCR6), activation markers (e.g. CD57, CD95), cytokines (IFNγ, TNFα, IL-2, IL-4, IL-17A) and lytic enzymes (perforin, granzyme B)^38^. Moreover, circulating DPT cells from rheumatoid arthritis patients produce both anti-inflammatory (IL4 and IL21) and pro-inflammatory (IFNγ) cytokines^39,40^. Additionally, DPT cells from the peripheral blood of patients with urologic cancer favor ex vivo polarization of naïve CD4+ T cells into Th2 cells^29^. Thus, kidney DPT cells appear to be a heterogenous population with both inflammatory and immunosuppressive functions.

Kidney DPT cells were also metabolically active at baseline and showed significantly increased GLUT1 expression following IR injury and LCMV infection. Activated T cells prefer glucose utilization and upregulate GLUT1 expression^41,42^. Other metabolic markers affected after IR and viral infection had different magnitude and directions. Interestingly, cisplatin-induced AKI and absence of bacteria in GF mice did not affect the expression of any of these metabolic markers in kidney DPT cells. These results may suggest that metabolic programing of kidney DPT cells varies depending on the type of injury.

We found that kidney DPT cells had elevated PD1 expression at baseline, which further increased after cisplatin-induced AKI and LCMV infection. Checkpoint inhibitory receptors such as PD1 are important for regulating T cell functions. Recent data from tumor-infiltrating lymphocytes (TILs) from RCC, lung, and colon cancer patients found very high PD-1 and TIM-3 expression on DPT cells^43^. These results suggest that DPT cells could be dysfunctional/exhausted following cytotoxic chemotherapy or viral infection.

Though we observed an effector/central memory profile and proinflammatory phenotype at baseline using flow cytometric approach, our scRNA-seq analysis of kidney DPT cells showed significant expression of *Sell, Tcf7* and *Il7r* genes which are associated with a naïve T cell phenotype. Additionally, scRNA-seq analysis showed increased expression of *Klf2* in DPT cells which is a transcriptional marker of naïve T cells and enhances their migration to non-lymphoid organs^44^. Consistent with our results, a recent study performing scRNA-seq in peripheral blood of cynomolgus monkeys found a significant naïve DPT cell subset expressing *Sell, Tcf7* and *Il7r*^45^. Future studies should determine the protein level of these naïve kidney DPT cell markers used for scRNA-seq data annotation to define their exact memory status.

Although this study provided initial characterization of kidney DPT cells during multiple different stimuli and conditions in both humans and mice, it lacks studies examining causal role of DPT cells in kidney diseases. However, technical limitations related to DPT cell depletion or enrichment for exploring their pathophysiologic functions pose a challenge, as traditional antibody depletion approach would also affect CD4 and CD8 T cells. Furthermore, this study does not provide information on the mechanism that regulate DPT proportions to such low levels. A significant limitation was that the extremely small numbers of kidney DPT cells prevented the feasibility of in vitro co-culture or adoptive transfer studies. Future studies, perhaps with more advanced technologies, are required to explore mechanisms that regulate DPT cell functions and interactions in kidney under normal conditions and during disease.

In summary, DPT cells account for a minor subset of kidney T lymphocyte and their frequency is augmented during ischemic and nephrotoxic AKI as well as LCMV infection. Kidney DPT cells are activated and have a memory profile with distinct cytokine expression and metabolic demands under normal and disease conditions. These findings on kidney DPT cells provide an important framework for future studies designed to further define the exact mechanisms that regulate kidney DPT cells during normal health and disease, and how kidney DPT cells participate in kidney diseases.

## Materials and methods

### Animals

8–10-week-old male C57BL/6 J wild type (WT) mice were procured from Jackson Laboratory (Bar Harbor, ME) and housed under specific pathogen-free conditions at the animal facility of the Johns Hopkins University. Germ-free (GF) mice were generated and maintained at the Johns Hopkins germ-free mouse core facility. The Johns Hopkins University Animal Care and Use Committee approved all experimental protocols described in this study. The data related to this study has been reported in compliance with Animal Research: Reporting of In Vivo Experiments (ARRIVE) guidelines^46^.

### Human samples

Human kidney tissue was obtained from patients undergoing nephrectomies due to renal cell carcinoma (RCC) and KMNCs were isolated following an established protocol. The staining protocol used for flow cytometric analysis of human samples was same as described for murine samples. Studies using human kidney tissue were performed in adherence to the Declaration of Helsinki. An informed consent was obtained as per approved protocol by the Johns Hopkins Institutional Review Board.

### Ischemic AKI model

Mice were anesthetized with sodium pentobarbital (75 mg/kg) via intraperitoneal (i.p.) injection. After a medial abdominal incision and renal pedicle dissection, a microvascular clamp (Roboz Surgical Instrument, Gaithersburg, MD) was placed on each renal pedicle for 27 min. Mice were hydrated with warm saline and maintained at a constant body temperature of 37 °C. Clamps were removed after 27 min of ischemia, wounds were sutured and the kidneys were allowed to reperfuse. For AKI to CKD studies, only the left kidney was clamped for 45 min, and DPT cells were studied 4 weeks later. Mice were provided free access to food and water after completion of the surgical procedure.

### Cisplatin-induced AKI model

Mice were injected a single intraperitoneal injection (25 mg/kg body wt) of freshly prepared cisplatin (cis-diammineplatinum II dichloride, Sigma-Aldrich, St. Louis, MO) dissolved in normal saline (1 mg/mL). Blood samples were collected by clipping the tail vein at baseline (0 h) and 24, 48, and 72 h after cisplatin injection. Serum creatinine (SCr) was measured with Cobas Mira Plus automated analyzer (Roche, Nurley, NJ) using the Creatinine reagent set (Pointe Scientific, Canton, MI).

### Lymphocytic choriomeningitis virus (LCMV) infection model

WT mice were infected with LCMV Armstrong virus (2 × 10^5^ pfu/mouse, i.p.) and kidneys were collected at the peak of infection, 7 days post-infection.

### Isolation of kidney mononuclear cells (KMNCs)

Kidney mononuclear cells (KMNCs) were isolated following a previously established protocol^47^. Briefly, mice were anesthetized intraperitoneally with ketamine (140 mg/kg) and xylazine (10 mg/kg) solution and exsanguinated to remove circulating lymphocytes from the kidneys. Kidneys were decapsulated, minced and incubated in collagenase D (2 mg/mL, Sigma-Aldrich) for 30 min at 37 °C. Subsequently, kidneys were mechanically disrupted using 70-μm strainers (BD Bioscience) and single cell suspensions were obtained. KMNCs were enriched using Percoll density gradient centrifugation, washed and counted before downstream flow analysis using Aurora spectral flow cytometer (Cytek Biosciences).

### Antibodies and reagents

Following fluorochrome-conjugated anti-mouse antibodies were used for analyzing T cell identification, activation status, cytokines profile and metabolism. All antibodies were procured from BioLegend unless specified otherwise. *T cell identification, activation status and cytokines panel:* Spark Blue 550 anti-CD45 (30.F11), Alexa Fluor 488 anti-TCRβ (H57-597), PerCP-Cy5.5 anti-CD8α (53–6.7), BV480 anti-CD4 (RM4-5), Alexa Fluor 700 anti-CD69 (H1.2F3), BV785 anti-CD62L (MEL-14), BV570 anti-CD44 (1M7), APC-Fire 750 anti-PD-1 (29F.1412), BV 421 anti-Ki67 (16A8), BV650 anti-IFNγ (XMG1.2), PerCP-eFluor anti-IL4 (11B11), PE-Dazzle 594 anti-IL10 (JES5-16E3), BV605 anti-IL17 (TC11-18H10.1) and BV711 anti-TNFα (MP6-XT22). *Metabolic panel:* Pacific blue anti-CD44 (IM7), BV510 anti-CD8 (53–6.7), BV570 anti-CD45 (30-F11), BV605 anti-CD69 (H1.2F3), BV650 anti-NK1.1 (PK136), BV711 anti-PD1 (29F.1A12), BV785 anti-TCRβ (H57-597), PE-Cy5.5 anti-CD25 (PC61.5, Thermo Fisher Scientific), APC-R700 anti-CD62L (MEL-14, BD Bioscience), and APC-Fire810 anti-CD4 (GK1.5), Alexa Fluor 488 anti-CPT1a (8F6AE9, Abcam), Alexa Fluor 532 anti-VDAC1 (20B12AF2, Abcam), PerCP-efluor 710 anti-FoxP3 (FJK-16S, Thermo Fisher Scientific), PE anti-GLUT1 (EPR3915, Abcam), Alexa Fluor 594 anti-pS6 (D68F8, Cell Signaling), PE-Cy5 anti-HKII (EPR20839, Abcam), PE-Cy7 anti-H3K27me3 (C36B11, Cell Signaling), and Alexa Fluor 647 anti-Tomm20 (EPR15581-54, Abcam).

### Human kidney flow panel

The following antibodies were used to identify T cell subsets in human kidney samples: BV570 anti-CD45RA (HI100), PE-Cy5 anti-CD4 (OKT4) and BV480 anti-CD8 (RPA-T8, BD Biosciences).

### Multiparameter spectral flow cytometry

Prior to staining, 1X10^6^ KMNCs/sample designated for intracellular cytokine analysis were stimulated with leukocyte activation cocktail containing phorbol 12-myristate 13-acetate (PMA), ionomycin and Brefeldin A (BD Biosciences), for 4 h at 37 °C in a 5% CO2 incubator. Otherwise, 1X10^6^ KMNCs/sample were washed with PBS to facilitate fluorochrome staining. Subsequently, cells were resuspended in 100 ul of diluted Zombie NIR (1:1000) and incubated for 20 min at room temperature. Cells were washed with PBS and incubated with anti-CD16/CD32 FC block (S17011E, BioLegend) for 15 min at 4 °C. A cocktail of surface staining antibodies was then added directly to cells and incubated for an additional 30 min. Following the surface staining protocol, cells were washed and incubated for 30 min at 25 °C with fixation and permeabilization solution. After another wash step, cell suspensions were incubated with the cocktail of intracellular staining antibodies for an additional 45 min. Cells were washed and resuspended in 150 ul of FACS buffer in preparation for sample reading with the Cytek Aurora Spectral Flow Cytometer.

### AMNIS single cell imaging flow cytometry

Freshly isolated KMNCs (1X10^6^) from normal mouse kidneys were stained with CD45 (APC-Cy7), TCR (BV 421), CD4 (Alexa Fluor 488) and CD8 (PE) antibodies and analyzed on AMNIS Imagestream-X MarkII Imaging Flow Cytometer (EMD Millipore). 7AAD was used to label and exclude dead cells from the analysis. Single color-stained spleen cells were used to compensate for fluorochrome spillover and data was analyzed using IDEAS (v6.2).

### Single-cell RNA seq

Single cell analysis of flow sorted kidney CD45+ cells was performed using a previously published dataset as described by Noel et al.^34^. The dataset used for this analysis is available at https://zenodo.org/record/7314511.

### Statistical analysis

Unmixed flow data files (.fcs) were analyzed using FlowJo (BD Biosciences software) as per the gating strategy shown in Fig. 1A. UMAP analysis of flow data was performed using UMAP plugin for FlowJo software. For metabolic marker analysis, raw MFI value (geometric mean) derived from FlowJo were normalized by subtracting minimum column value and dividing by the difference between column maximum and column minimum values. ((MFI value–column minimum)/(column maximum–column minimum)). Comparisons between two groups were conducted using non-parametric Mann–Whitney t-tests. For comparisons including more than two groups, one-way ANOVA Kruskal–Wallis test was performed followed by Dunn’s multiple comparisons post hoc test. Data was represented as mean ± SEM using GraphPad Prism (10.0.2), where n indicated the number of mice per group. *P* value less than or equal to 0.05 was interpreted as statistically significant difference between the groups.

### Supplementary Information

Supplementary Figures.

## Data Availability

scRNA-Seq data used in this study has been deposited at Zenodo, a data repository of CERN and can be accessed at 10.5281/zenodo.7314510.
