# Supplementary material for: Kidney double positive T cells have distinct characteristics in normal and diseased kidneys
Source: Sci Rep. 2024 Feb 23;14:4469. doi: 10.1038/s41598-024-54956-3 (PMC10891070; doi:10.1038/s41598-024-54956-3)
Supplement: Supplementary file 1 — Supplementary Figures. [file 41598_2024_54956_MOESM1_ESM.pdf]

Supplemental table of contents

Supplemental figure 1. Analysis of splenic DPT cells at baseline and comparison with kidney DPT cells.

Supplemental figure 2. Kidney DPT cell analysis after cisplatin treatment.

Supplemental figure 3. Kidney DPT cell activation and metabolic changes following LCMV infection.

Supplemental figure 4. Effect of gut microbiota on kidney DPT cells proportion, activation and metabolism.

Supplemental figure 5. Kidney DPT cell proportion and cytokine profile, 4 weeks post IRI in severe AKI to CKD model.

Supplemental Figure 1

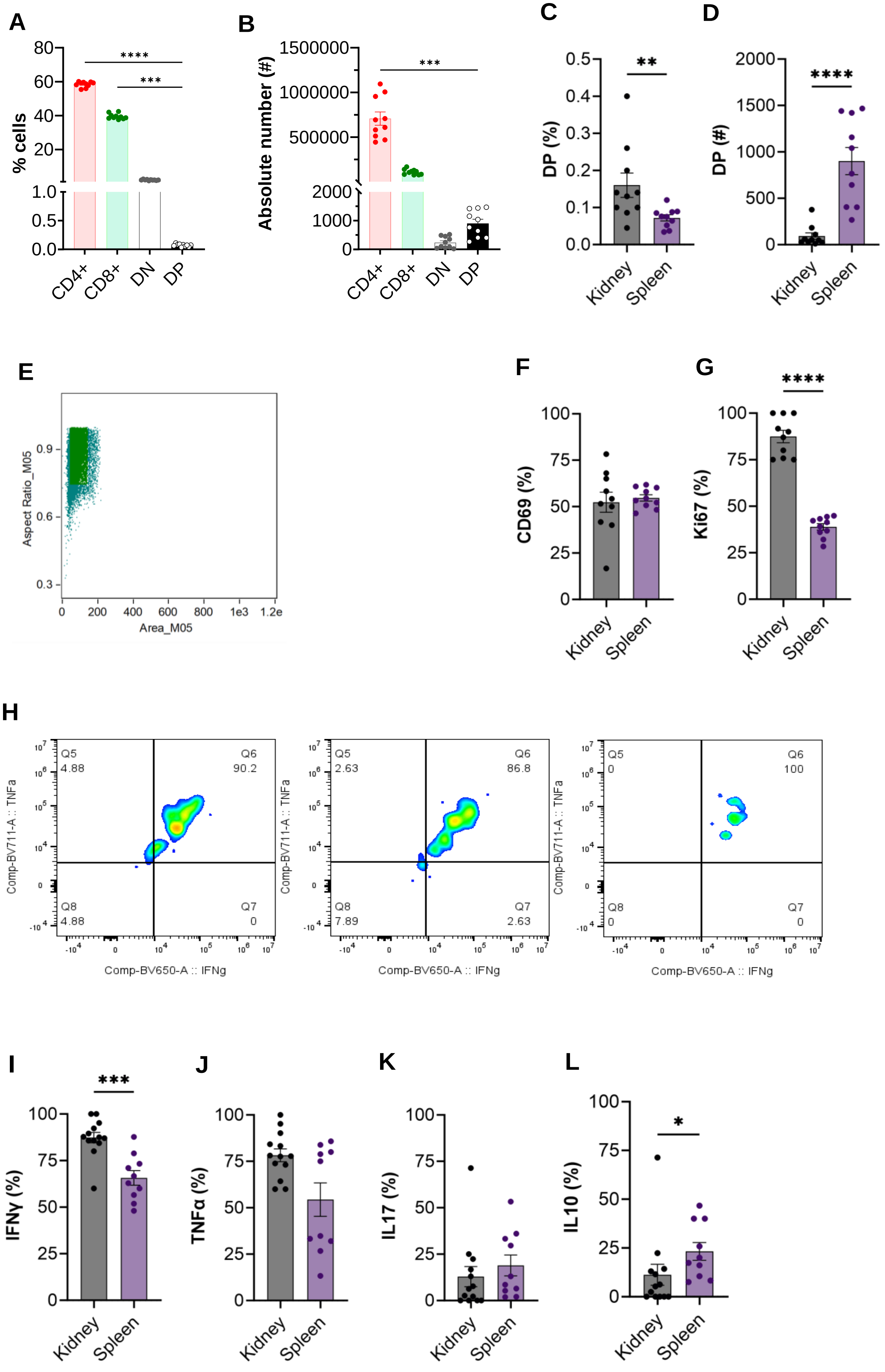

Supplemental figure 1. Analysis of splenic DPT cells at baseline and comparison with kidney DPT cells. Graphs showing percentage (A) and absolute number (B) of splenic DPT cells compared to CD4, CD8 and DNT cells at baseline. Percentage (C) and absolute number (D) of kidney DPT cells compared to spleen at baseline. (E) Area and aspect ratio plot showing single cells in dark green. Percent of kidney DPT cells expressing activation markers CD69 (F) and Ki67 (G) compared to splenic DPT cells. (H) Representative flow dot plots showing IFNγ+TNFα+ DP T cells. Graphs showing intracellular cytokines IFNγ (I), TNFα (J), IL17 (K) and IL10 (L) in kidney DP T cells compared to splenic DPT cells. Data are expressed as mean±sem and compared by non-parametric Kruskal-Wallis test followed by Dunn's post-hoc analysis. * = $p \leq 0.05$, ** = $p \leq 0.01$, *** = $p \leq 0.001$, **** = $p \leq 0.0001$.

Supplemental Figure 2

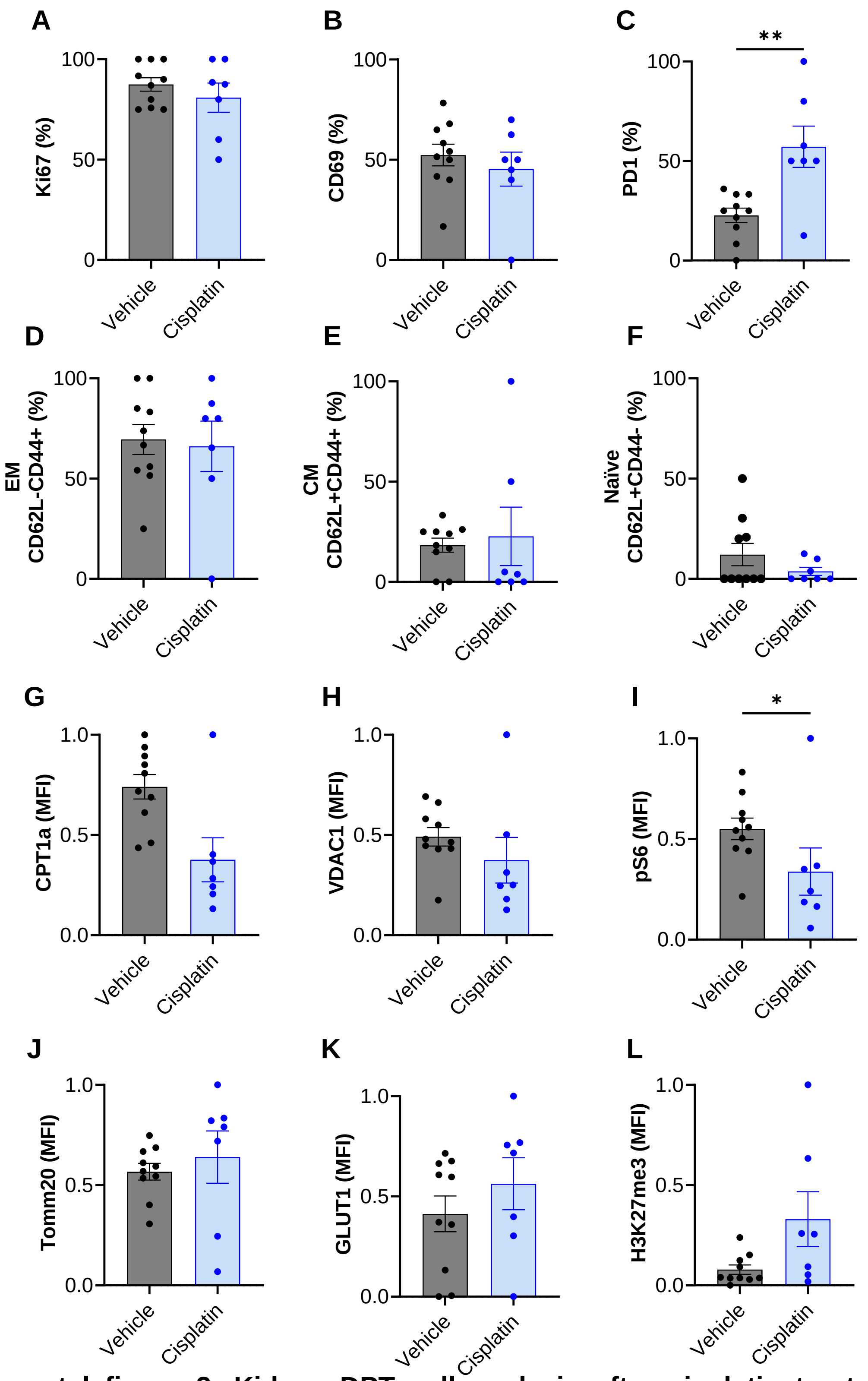

Supplemental figure 2. Kidney DPT cell analysis after cisplatin treatment. Graphs showing the percentage of kidney DPT cell expressing proliferation marker Ki67 (A), activation marker, CD69 (B) and immune checkpoint molecule PD1 (C) in vehicle and cisplatin treated mice. (D-F) Memory profile of kidney T cells showing EM, CM and naïve DPT cells in vehicle and cisplatin treated mice. (G-L) Graphs showing mean fluorescent intensity (MFI) of metabolic markers CPT1a, VDAC1, pS6, Tomm20, GLUT1 and H3K27me3 in kidney DPT cell from vehicle and cisplatin treated mice. Data are expressed as mean±sem and compared by non-parametric Kruskal-Wallis test followed by Dunn's post-hoc analysis. * = p≤0.05, ** = p≤0.01.

Supplemental Figure 3

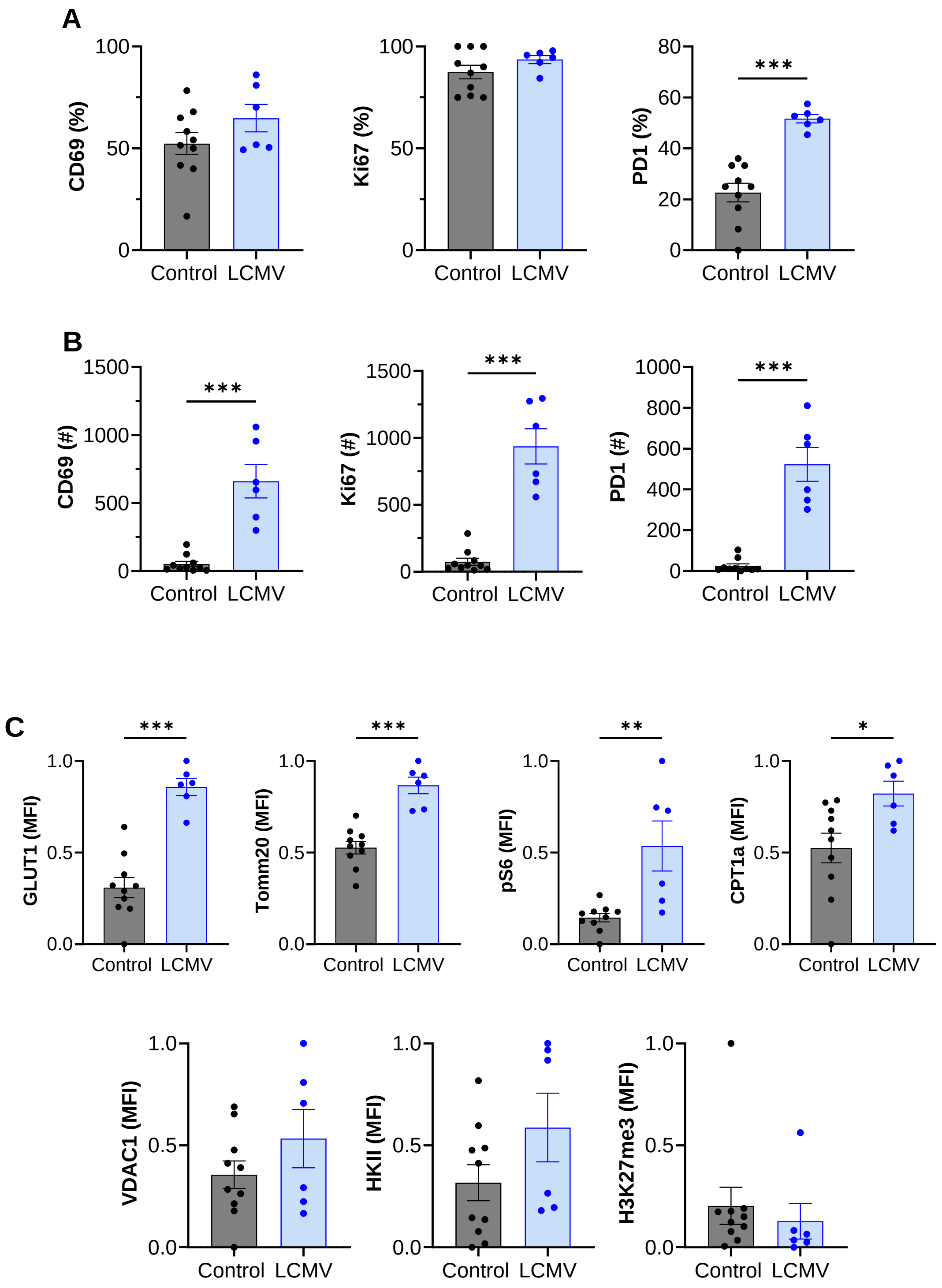

Supplemental figure 3. Kidney DPT cell activation and metabolic changes following LCMV infection. (A) Graphs showing percentage (upper) and (B) absolute number (lower) of activation marker CD69, proliferation marker Ki67 and immune checkpoint molecule PD1 in kidney DPT cell from LCMV infected and control mice. (C) Graphs showing mean fluorescent intensity (MFI) of metabolic markers GLUT1, Tomm20, pS6, CPT1a, VDAC1, HKII and H3K27me3 in kidney DPT cell from control and LCMV infected mice. Data are expressed as mean±sem and compared by non-parametric Kruskal-Wallis test followed by Dunn's post-hoc analysis. * = p≤0.05, ** = p≤0.01, *** = p≤0.001.

Supplemental Figure 4

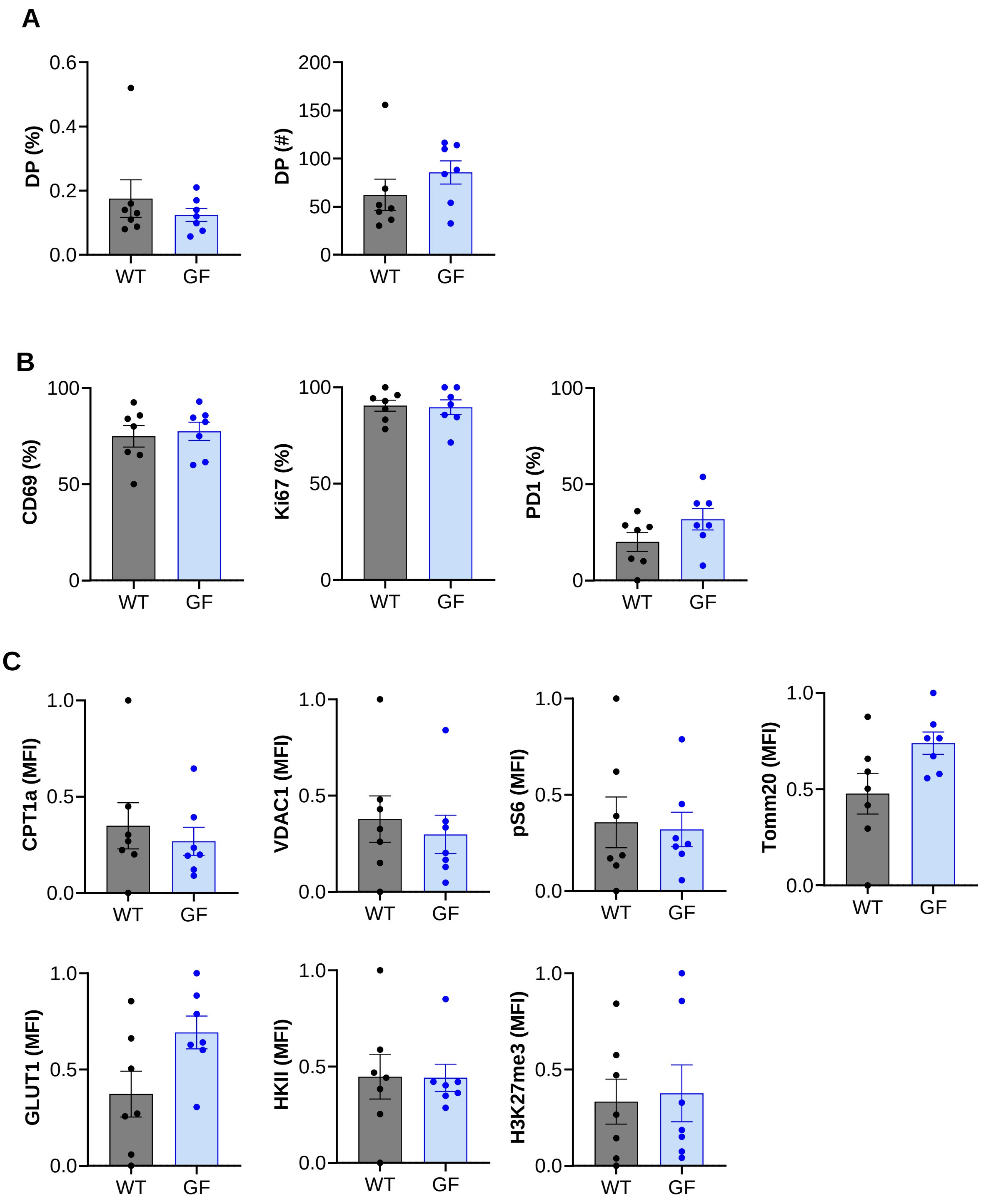

Supplemental figure 4. Effect of gut microbiota on kidney DPT cells proportion, activation and metabolism. (A) Graphs showing percentage (left) and absolute number (right) of kidney DPT cells in WT control and GF mice. (B) Graphs showing percentage of activation marker CD69, proliferation marker Ki67 and immune checkpoint molecule PD1 in kidney DPT cells from WT control and GF mice. (C) Graphs showing mean fluorescent intensity (MFI) of metabolic markers CPT1a, VDAC1, pS6, Tomm20, GLUT1, HKII and H3K27me3 in kidney DPT cell from WT control and GF mice. Data are expressed as mean±sem and compared by non-parametric Kruskal-Wallis test followed by Dunn's post-hoc analysis.

Supplemental Figure 5

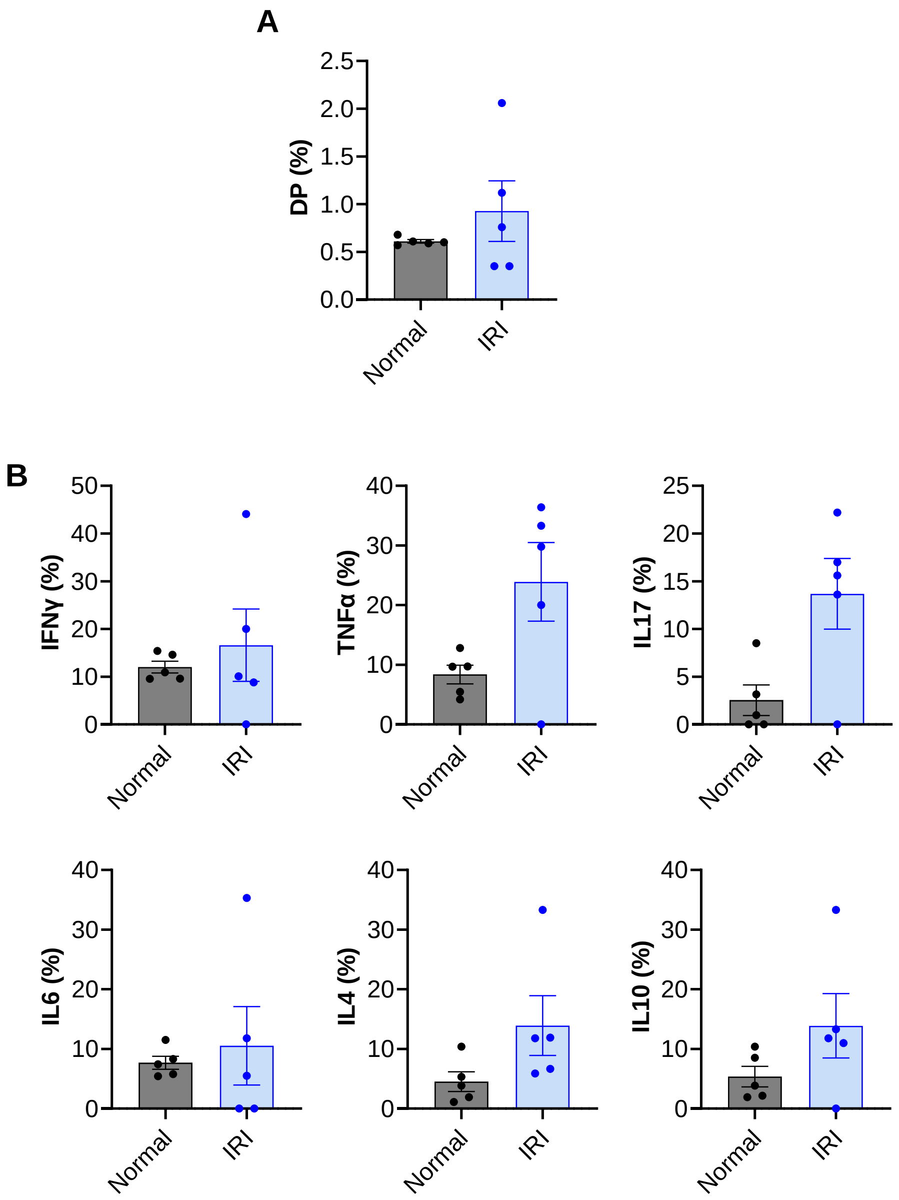

Supplemental figure 5. Kidney DPT cell proportion and cytokine profile, 4 weeks post IRI in severe AKI to CKD model. (A) Graph showing the percentage of kidney DPT cells in normal contralateral and ischemic kidneys of mice that underwent unilateral severe IRI to model AKI to CKD transition. (B) Percent of kidney DPT cells expressing intracellular cytokines IFNγ, TNFα, IL17, IL4, IL6 and IL10 in normal contralateral and ischemic kidneys of mice that underwent unilateral severe IRI. Data are expressed as mean±sem and compared by non-parametric Kruskal-Wallis test followed by Dunn’s post-hoc analysis.
